# A single centre experience of squamous cell carcinoma of the upper limb requiring digital or hand amputation and review of literature

**DOI:** 10.1016/j.jpra.2019.01.001

**Published:** 2019-01-09

**Authors:** Leela Sayed, Avinash K. Deodhar, Reena Agarwal

**Affiliations:** Department of Plastic Surgery, University Hospitals of Leicester, Infirmary Square, Leicester LE1 5WW, United Kingdom

**Keywords:** Squamous cell carcinoma, Upper limb, Upper extremity, Hand, Digits

## Abstract

Squamous cell carcinoma (SCC) is one of the most common primary malignancies affecting the upper limb. A range of treatment options exist for its management; amputation being indicated under certain instances. This is the first comprehensive case series and review of the literature reporting outcomes following amputation of the affected region for treatment of upper extremity SCC**.** We present a series of six patients with squamous cell carcinoma of the upper limb that required amputation alongside that of data from literature review. Patient demographics, risk factors, tumour characteristics and rates of recurrence, metastasis and mortality were recorded. A total of 45 patients with 49 histologically confirmed squamous cell carcinomas were identified from case series and literature review. Patients presenting with upper limb SCC were predominantly male and in their sixth decade of life. Mean follow up time was 30.5 months and the overall recurrence and metastatic rates were 8.2% and 14.3%, respectively. Mortality was 14.3% however only 6.1% was related to SCC metastasis. Rates of recurrence and metastasis are higher for SCCs affecting the hand as compared to other body sites. Furthermore, different regions of the hand appear to behave differently. SCC affecting the nail unit has a high recurrence and a low metastatic rate, whereas, SCC involving the palm and webspaces are aggressive and this is true despite amputation of the affected site.

## Introduction

Non-melanoma skin cancer (NMSC) accounts for approximately 90% of all skin cancers registered in the UK and Ireland with squamous cell carcinoma (SCC) representing around 23% of these^1^. Furthermore, it is the most common primary malignant tumour of the hand^2^ and studies of full body anatomical distribution report it to be present on the upper limb in 9-35% of cases^3^, ^4^, ^5^. When comparing relative tumour densities of other NMSCs such as basal cell carcinoma (BCC), the most notable difference was the prevalence in the hand with a BCC:SCC ratio of 1:14.^5^

Ultraviolet radiation is the most common cause of cutaneous SCC in the Caucasian population and produces mutations in the P53 tumour suppressor gene.^6^ Occupational and environmental exposure to radiation, arsenic and polycystic hydrocarbons are well known carcinogens^7^ and more recently Human Papilloma Virus (HPV) infection has been shown to have oncogenic potential.^8^, ^9^. Patients that are immunosuppressed either via an iatrogenic or a disease-causing process are also at higher risk of developing SCC.^10^ Other risk factors include genodermatoses such as xeroderma pigmentosum and in injured or chronically inflamed skin such as burns, ulcers and cutaneous manifestations of lupus and lichenoid disease.

Cutaneous SCC arises following malignant transformation of epidermal keratinocytes. Histopathological features include nuclear atypia, pleomorphic keratinocytes, hyperkeratosis and parakeratosis. When confined to defined foci within the epidermis, actinic keratosis; a precursor of squamous cell carcinoma is present. Carcinoma in-situ or Bowen's disease displays the above features but constitutes epidermal full thickness involvement. Invasion of the basement membrane of these atypical cells surmounts to invasive SCC and can be divided into well, moderately and poorly differentiated disease based on the degree of nuclear atypia, keratinisation and frequency of mitoses.

In general, invasive SCC has a 5-year recurrence and metastasis rate of 8% and 5%, respectively.^11^, ^12^ However, numerous studies have reported a higher rate of recurrence and metastasis for tumours occurring on the hand and in particular those arising from the dorsal surface and webspaces.^13^, ^14^, ^15^

Treatment of upper extremity SCC ranges from shave excision, curettage and cautery to cryotherapy for small low risk lesions to Moh's micrographic surgery, wide local excision and finally amputation. In general radiotherapy is not used for curative local control of SCC of the hand due to high rates of radionecrosis; however, it can be useful for patients that are not suitable for surgical excision. Indications for amputation include massive soft tissue involvement, bony infiltration, patient preference and contraindications for reconstructive surgery. To our knowledge this is the first case series of 6 patients and comprehensive literature review of 49 upper limb SCCs requiring amputation. Other facets for consideration of amputation are discussed in conjunction with current literature.

## Patients and methods

A single centre, retrospective case note analysis of 6 patients presenting with SCC of the hand requiring amputation was undertaken between 2013 and 2018. A comprehensive literature review was also performed using Ovid MEDLINE, Pubmed and Google Scholar using the following key words/phrases: Squamous cell carcinoma, upper limb, upper extremity, hand, digits. References from articles raised from the search were also considered to expand literature review.

Patient demographics, risk factors, tumour site and length of time from symptoms to diagnosis of SCC were recorded. Grade of tumour was documented as: well differentiated, moderately differentiated and poorly differentiated. In cases where a single lesion was reported to have different grades, the highest grade was recorded. Additionally, it was presumed that documentation of a ‘differentiated’ lesion to be a well differentiated SCC. Level of amputation was defined as the definitive procedure resulting in tumour clearance whether that be a single procedure or multiple procedures for involved margins or recurrence. Length of follow-up, recurrence, metastasis and mortality were also recorded. Length of follow-up was recorded as time from amputation to last clinic review. Recurrence was documented as any SCC arising at or adjacent to the site of the primary lesion where histological clearance was previously achieved.

## Case series

**Figure 1:**
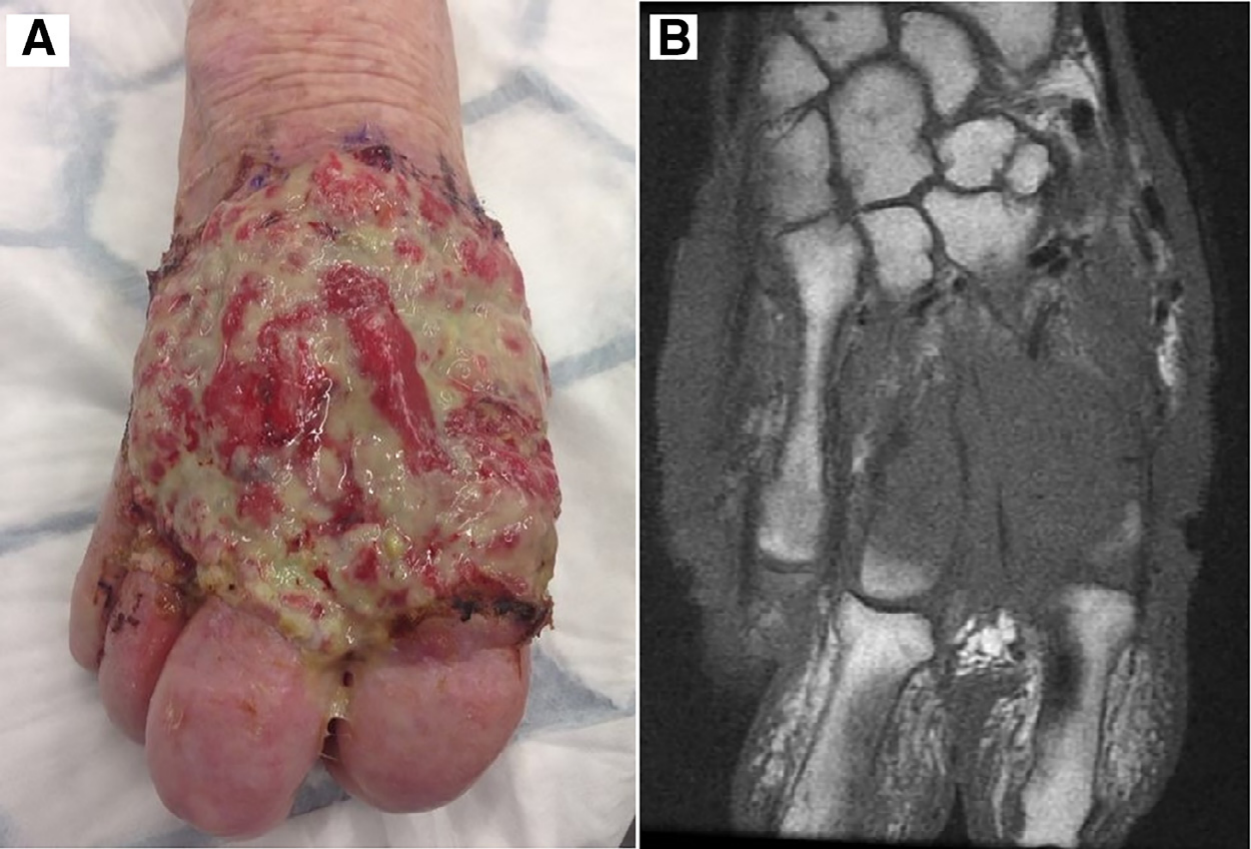
(A) Showing SCC involving dorsum of right hand for Case 1. (B) MRI showing tumour extension into index and middle metacarpal bones.

*Case 1.* An 84-year-old female presented with a 5 year history of a fungating lesion on the dorsum of her right hand. The lesion was large and fixation to underlying structures was reported; no axillary lymphadenopathy was present. A biopsy, not unexpectedly reported a poorly differentiated, 13.7mm thick SCC and MRI revealed extensive involvement of the second and third metacarpals and interossei which extended volarly. CT chest, abdomen and pelvis showed no evidence of metastatic spread. Following MDT and patient discussion a distal forearm amputation was undertaken. Recovery was unremarkable and there has been no recurrence or metastatic spread since last clinical review at 48 months (Figure 1).

**Figure 2:**
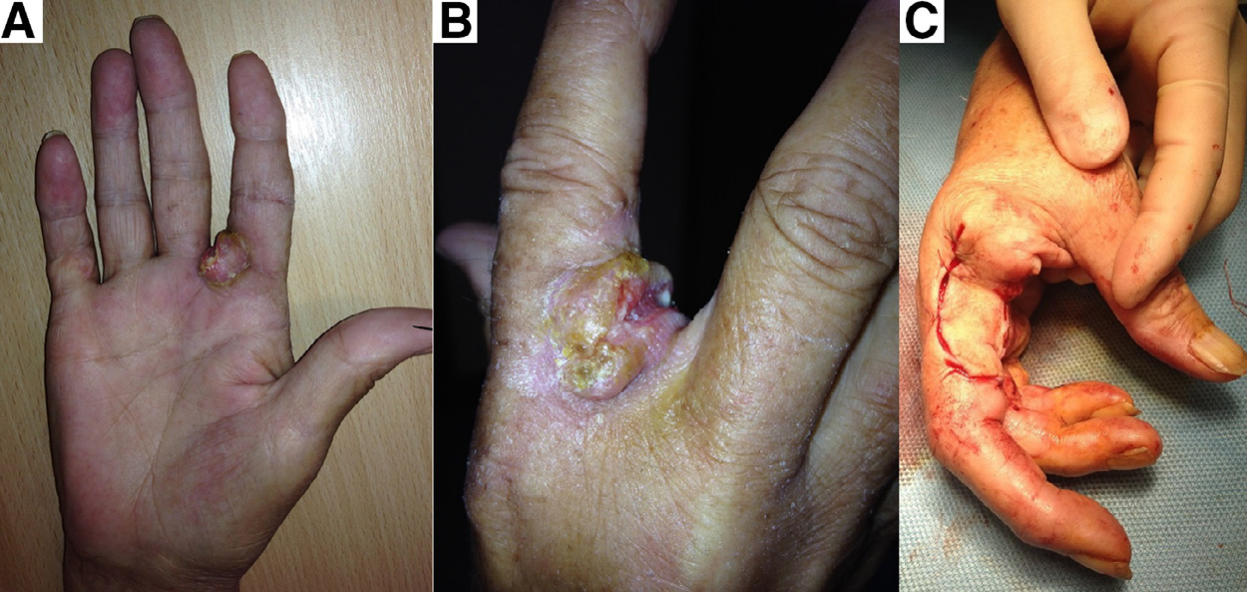
(A), (B) Showing volar, dorsal and webspace involvement of SCC. (C) Showing amputation of right index finger at the level of MCPJ and radially based skin flap from right index finger.

*Case 2.* A 71-year-old female was referred from Dermatology with a 5-month history of a fixated swelling on the right index finger and an ulcerated lesion involving the second webspace; a biopsy showed this to be a well differentiated SCC. There was no clinical or radiological evidence of lymphatic or metastatic spread. The patient underwent amputation of the right index finger at the level of the metacarpal phalangeal joint (MCPJ) under general anaesthesia. Histological analysis reported a completely excised moderately differentiated SCC measuring 9.9mm thick. She was discharged home the same day and 48 months later has had no sign of recurrence or metastasis (Figure 2).

*Case 3.* A 67-year-old male was referred by a Dermatologist with a 2-month history of biopsy proven squamous cell carcinoma present on each hand. The lesion on the right hand involved the middle finger and third webspace and the lesion on the left hand involved only the dorsum. There was no sign of axillary lymphadenopathy bilaterally. This gentleman suffered from COPD, had a tracheostomy in situ for previous T4 vocal cord tumour and was a heavy smoker.

**Figure 3:**
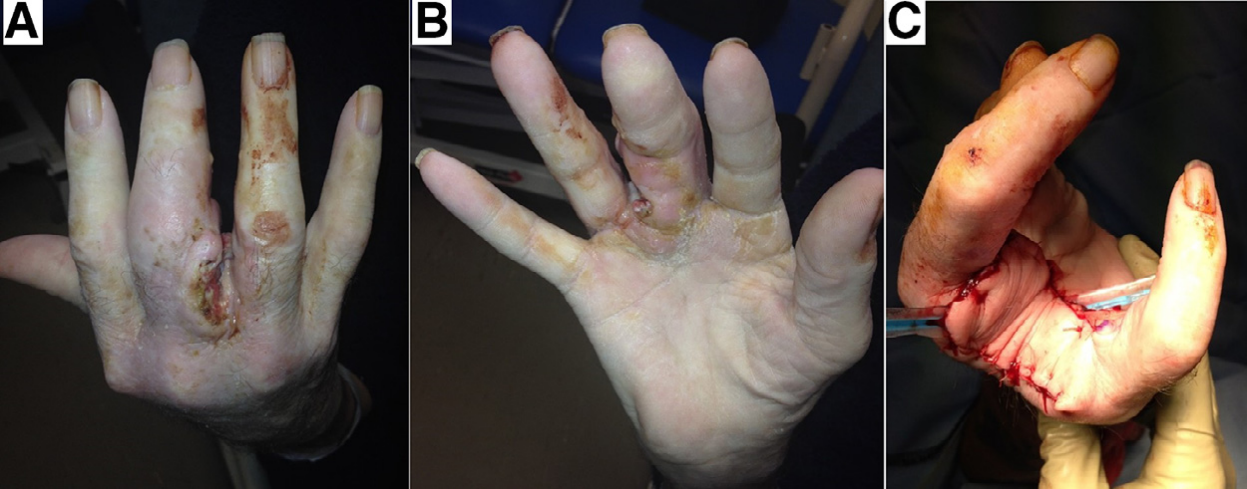
(A), (B) Showing volar, dorsal and webspace involvement of SCC. (C) Showing amputation of right middle and ring fingers at the level of MCPJ.

He underwent amputation of the right middle and ring fingers at the level of the MCPJ and was reconstructed using a fillet flap from ring finger. Histology reported a moderately differentiated, 17 mm thick SCC. His recovery was unmarkable and recurrence was not detected within the first post-operative year. Unfortunately, he died following sequelae of the vocal cord tumour (Figure 3).

**Figure 4:**
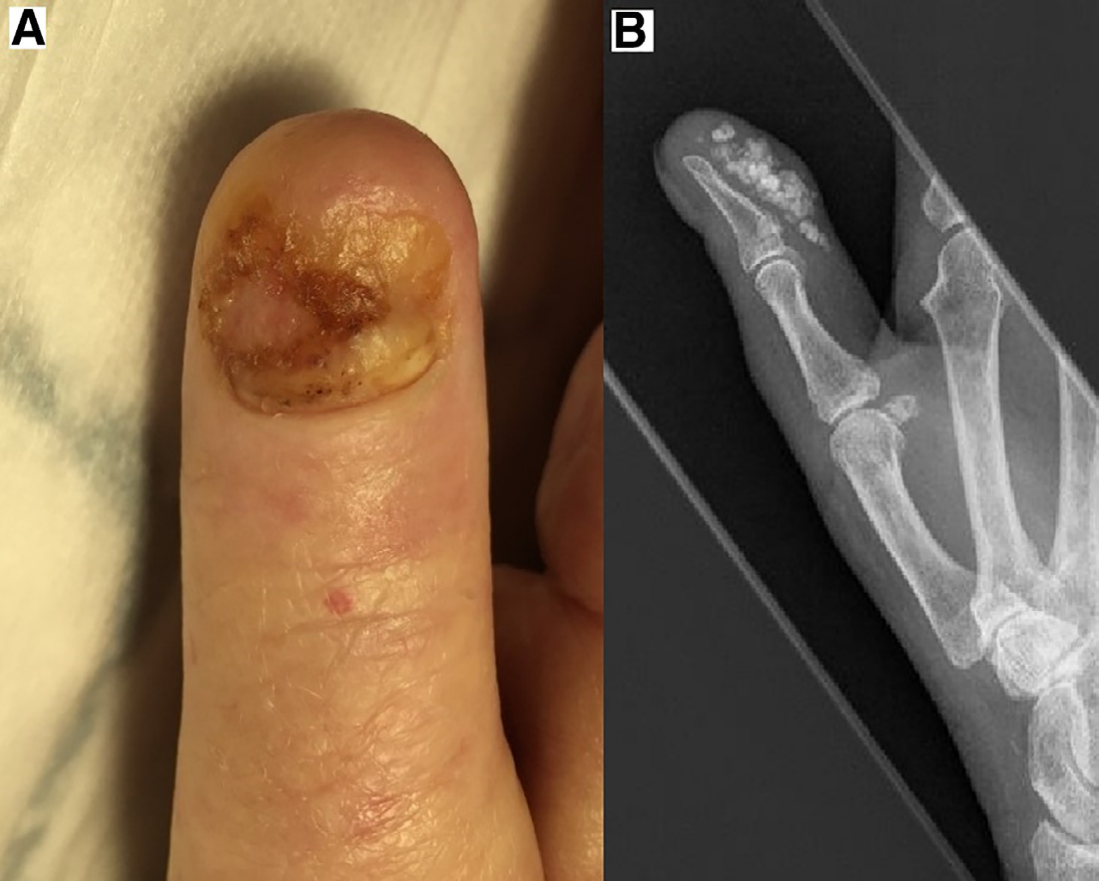
(A) Showing right thumb nail bed SCC. (B) Radiograph of right thumb showing extensive subcutaneous calcinosis.

*Case 4.* A 75-year-old female with a background of scleroderma, Raynaud's disease, hypothyroidism and type 2 diabetes was referred via her rheumatologist for pain and onycholysis of the right thumb. On examination the underlying nail bed was unremarkable aside from some variation in colour; there was no axillary lymphadenopathy. Surprisingly a biopsy of the nail bed revealed a 3.3mm thick moderately differentiated SCC. Following MDT discussion, the patient underwent amputation of the distal phalanx under local anaesthetic. The stump healed well and has showed no evidence of recurrence or metastasis at last clinic review 22 months later (Figure 4).

*Case 5.* A 78-year-old female presented with a 4-month history of a crusted nail bed lesion and nail plate dystrophy of the right index finger; there was no axillary lymphadenopathy. She also suffered from type 2 diabetes, gout and hypertension. Histology revealed a moderately differentiated SCC arising from bowenoid dysplasia. Following MDT discussion, she underwent right index finger amputation through the DIPJ using a volar skin flap for coverage under local anaesthetic. The 2.3 mm thick SCC was completely excised however Bowen's disease was present at the lateral margin and was therefore treated topically. She has had no sign of recurrence or metastasis for 14 months.

**Figure 5:**
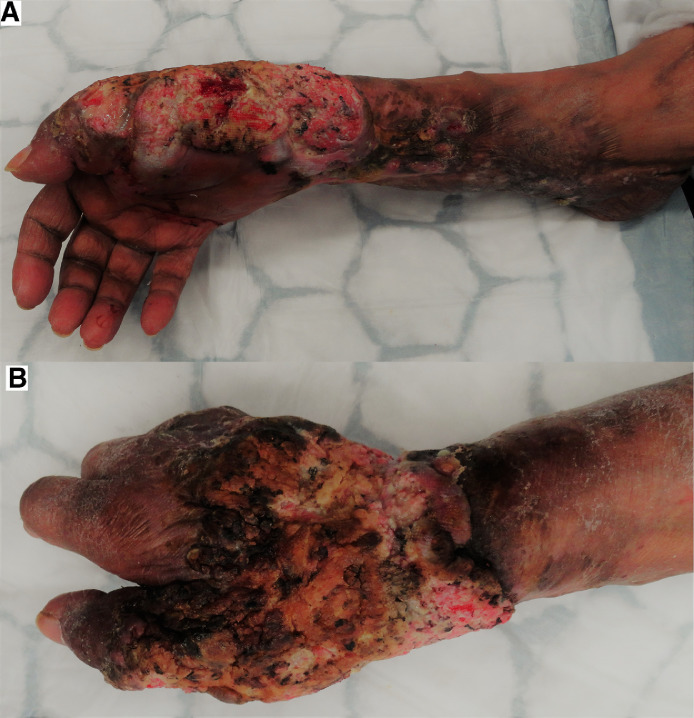
(A), (B) Showing volar and dorsal involvement of SCC on right upper limb.

*Case 6.* An 84-year-old female presented to the Plastic Surgery Department following a non-healing ulcer on her right hand, wrist and forearm. She sustained a hot oil scald to both upper limbs at the age of 23 whilst living in Kenya. The burns were excised and resurfaced with a split thickness skin graft. Unfortunately, she developed an unstable scar on the right upper limb since her injury and has been managed with dressings and antibiotics. Skin biopsies were performed intermittently over the years which revealed inflammatory changes only, however, 6 years since the last skin biopsy, a 7mm thick well differentiated SCC was reported. The patient had one palpable lymph node in the right axilla however fine needle aspiration cytology and CT showed no evidence of metastasis. Due to the extensive nature of ulceration and poor skin quality of the adjacent areas it was decided that amputation of the hand at the level of the mid forearm was most appropriate. She has healed well and has showed no sign of recurrence nor metastasis 1 month post-operatively (Figure 5).

## Results

There were six patients with histologically confirmed SCC of the upper limb requiring amputation (Table 1). The six patients that underwent amputation had a mean age of 76.7 years (range, 67–84 years); 5 were female (83%) and 1 was male (17%). Tumour sites included 2 nail units (33.3%), 2 dorsum of hand and webspace (33.3%) and 1 dorsum of hand, webspace and palm (16.7%), 1 dorsum of hand, palm, wrist, forearm (16.6.%). Mean time from symptoms to diagnosis of squamous cell carcinoma was 29.8 months (range, 1–72 months). Five patients were Caucasian and reported UV exposure in the absence of sun protective measures; quantifying extent of exposure was however difficult to ascertain. One patient was a smoker and another patient had scleroderma and was on immunosuppressant therapy. One patient had previously treated Bowen's disease at a different site on the upper limb. There was a single patient of Indian origin who sustained a burn 60 years prior to development of an extensive SCC within the burn scar. Four SCCs were reported as moderately differentiated (66.7%), one was well differentiated (16.7%) and the other was poorly differently (16.7%).

**Table 1:** Patient demographics, tumour characteristics and outcomes from case series.

Case	Age (Years)	Sex Male (M)/Female (F)	Site Right (R)/Left (L)	Symptoms – diagnosis (Months)	Risk factors	Grade Of SCC	Level of amputation	Follow up (months)	Recurrence Yes (Y)/No (N)	Metastasis Yes (Y)/No (N)	Mortality Alive/Dead
1.	85	F	Dorsum R Hand, webspace, palm	60	UV exposure, Caucasian	Poorly differentiated	Distal forearm	48	N	N	Alive
2.	71	F	Dorsum R index 2nd webspace	5	UV exposure, Caucasian	Moderately differentiated	R 2nd MCPJ	48	N	N	Alive
3.	67	M	Dorsum R middle finger, 3rd webspace	2	UV exposure, Caucasian Smoker	Moderately differentiated	R 2nd – 3rd MCPJ	15	N	N	Dead Vocal cord tumour
4.	75	F	R Thumb nail bed	36	UV exposure Caucasian Scleroderma Immunosuppressants	Moderately differentiated	R thumb IPJ	22	N	N	Alive
5.	78	F	R index nail bed	4	UV exposure Caucasian Previous Bowen's Disease on R hand	Moderately differentiated	R index DIPJ	14	N	N	Alive
6.	84	F	R hand dorsum/palm, wrist, forearm	72	Burn to upper limbs/Marjolin ulcer	Well differentiated	R mid forearm	1	N	N	Alive

MCPJ – Metacarpal phalangeal joint; IPJ – Interphalangeal Joint; DIPJ – Distal Interphalangeal Joint.

There was one amputation through the DIPJ (16.7%), one through the IPJ (16.7%), two through the MCPJ (33.3%), one through the distal forearm (16.7%) and one through mid-forearm (16.7%). Mean length of follow up was 24.7 months (range, 1–48 months). No patient had recurrence or metastasis. One patient died, however this was unrelated to the cutaneous SCC as he had metastatic spread of a pre-existing vocal cord tumour.

## Discussion

Collation of data from case series and literature review yielded 59 patients with 63 lesions, however 14 patients with 14 lesions were excluded due to lack of follow-up data. Among the remaining 45 patients, 49 SCCs were confirmed from histopathological analysis (Tables 1 and 2).^16^, ^17^, ^18^, ^19^, ^20^, ^21^, ^22^, ^23^, ^24^, ^25^, ^26^, ^27^, ^28^, ^29^, ^30^, ^31^, ^32^, ^33^, ^34^, ^35^, ^36^, ^37^, ^38^, ^39^, ^40^, ^41^, ^42^, ^43^, ^44^ There were 27 males (60%) and 18 females (40%) with a mean age of 63.5 years (range, 23–92 years). This is in keeping with data from other studies reporting a male predominance and a peak incidence of SCC diagnosis in the sixth decade of life.^5^, ^13^, ^14^

**Table 2:** Patient demographics, tumour characteristics and outcomes from literature review.

Case	Reference	Age (Years)	Sex Male (M)/Female (F)	Site Right (R)/Left (L)	Symptoms – diagnosis (Months)	Risk factors	Grade Of SCC	Level of amputation	Follow up (months)	Recurrence Yes (Y)/No (N)	Metastasis Yes (Y)/No (N)	Mortality Alive/Dead
1	Abner et al, 2011^16^	46	F	(1) L thumb nail bed (2) L index nail bed (3) L ring nail bed (4) R middle finger nail bed	ND	Smoker	ND	Distal phalanx L thumb DIPJ L index, L ring, R middle fingers	12	N	N	Alive
2	Agir et al, 2007^17^	74	M	R palm, 2nd webspace, base of index	ND in English.	ND	Moderately differentiated	R index, middle MC shaft	10	Y	Y Radiotherapy	Dead Metastatic SCC
3	Alam et al, 2003^18^	78	M	R index dorsal DIPJ, proximal nail fold	ND	Previous BCC, actinic keratoses HPV -16 infection Previous SCC (same site)	Moderately differentiated	R index MCPJ	ND	Y	Y	Dead Metastatic SCC (Presumed)
4	Alghamdi et al, 2016^19^	63	M	(1) L thumb nail bed (2) L index Nail bed	22	Industrial worker (chemical exposure unknown)	1. ND 2. ND	1. L thumb IPJ 2. L index mid middle phalanx	86	N	N	Alive
5	Batalla et al, 2014^20^	55	M	L thumb nail bed	48	HPV	ND	L thumb IPJ	18	N	N	Alive
6	Batalla et al, 2014^20^	77	M	L thumb nail bed	24	Nil	Moderately differentiated	L thumb IPJ	30	N	N	Alive
7	Batalla et al, 2014^20^	79	M	L little finger nail bed	82	Nil	Well differentiated	L little DIPJ	26	N	N	Alive
8	Bosch et al 1999^21^	30	M	Dorsum R hand	6	Recessive dystrophic epidermolysis bullosa	Moderately differentiated	Distal 1/3 forearm	9	N	Y	Dead Metastatic SCC
9	Cardin-Langlois, 2010^22^	23	F	R palm, 2/3rd webspace, volar index and middle fingers	6	Kindler syndrome Previous verruciform actinic keratosis	Moderately differentiated	Below elbow	ND	Y	Y Axillary lymphadenectomy	ND
10	Chourghri et al, 2011^23^	54	F	L thumb nail bed	9	Trauma, chronic inflammation to digit	Well differentiated	L thumb IPJ	24	N	N	Alive
11	Figus et al, 2006^24^	25	M	Index, Lateral nail fold	10	Trauma	ND	Index DIPJ	36	N	N	Alive
12	Fino et al, 2015^25^	87	M	R dorsum hand, 2nd–3rd webspace and palm	48	Previous SSC (WLE x3 for incomplete excision) Radiotherapy to hand Chronic lymphocytic leukaemia (steroid therapy)	Moderately differentiated	Distal 1/3 forearm	ND	N	N	Alive
13	Fisher et al 2006^26^	33	M	R index finger, 2nd webspace, palm	17	Trauma Chronic infection Smoker	Well differentiated	R index / middle Metacarpal base	48	N	N	Alive
14	Foley et al, 1995^27^	52	M	L ring pulp	ND	Ionising radiation (dentist)	ND	L ring, mid middle phalanx	72	Y	Y Axillary lymphadenectomy Patient had adjuvant chemo radiotherapy	Alive
15	Gonzalez-Sosa et al, 2014^28^	63	M	L palm	96	Trauma Chronic infection	Moderately differentiated	L middle, ring, little CMCJ	1.5	N	Y Axillary lymphadenectomy	Alive
16	Grootenboers et al, 2007^29^	78	M	R ring nail bed	ND	ND	ND	R ring DIPJ	30	N	N	Dead Unrelated cause
17	Inkaya et al 2015^30^	84	F	L middle finger nail bed	12	Nil	Differentiated ‘Presumed well’	L middle finger PIPJ	12	N	N	Alive
18	Obiamiwe, 2001^31^	49	M	R thumb nail bed	63	Caucasian	ND ‘Invasive’	R thumb IPJ	ND	N	N	Alive
19	Ogawa et al, 2006^32^	60	M	R elbow, forearm	120	Trauma – Marjolin's ulcer Immunocompro-mised (Hep B/C, IVDU)	Moderately differentiated	R mid shaft humerus	5	N	N	Alive
20	Olaoye, 2013^33^	78	M	L thumb nail bed	108	ND	ND	L thumb IPJ	36	N	N	Alive
21	Peterson et al, 2004^34^	92	F	L thumb nail fold	ND	ND	Well differentiated	L thumb distal phalanx	38	N	N	Alive
22	Peterson et al, 2004^34^	88	F	R ring finger nail bed	ND	ND	Well differentiated	R ring finger DIPJ	17	N	N	Alive
23	Peterson et al, 2004^34^	43	M	R middle finger nail bed	ND	Previous SCC	ND	R middle finger DIPJ	15	N	N	Alive
24	Sakamoto et al, 2015^35^	41	M	R thumb pulp	0.5	ND	ND	R thumb IPJ	36	N	N	Alive
25	Sanchez et al, 2014^36^	89	M	R thumb nail bed	24	ND	ND	R thumb IPJ	ND	N	N	Alive
26	Shapiro and Baraf, 1970^37^	78	M	R ring finger nail bed	24	Trauma	ND – (Invasive)	R middle finger mid middle phalanx	48	N	N	Alive
27	Shapiro and Baraf, 1970^37^	70	M	L thumb nail bed	12	Nil	ND	L thumb IPJ	9	N	N	Alive
28	Shapiro and Baraf, 1970^37^	68	F	L Dorsum ‘index/middle fingers’ 2 webspace in a region of symbrachydactyly	ND	Nil	ND	L index, middle MCPJs	108	N	Y Axillary lymphadenectomy	Alive
29	Shapiro and Baraf, 1970^37^	68	M	R index nail bed	204	Caucasian	ND	R index PIPJ	2	N	N	Dead pulmonary embolism
30	Shapiro and Baraf, 1970^37^	65	M	L thumb nail bed	12	Caucasian	ND	L thumb proximal phalanx	15	N	N	Alive
31	Tabesh et al, 2003^38^	65	F	R index nail bed	240	Caucasian	ND	L thumb IPJ	36	N	N	Alive
32	Tambe et al, 2017^39^	63	M	L thumb nail bed	108	Trauma Infection	ND	L thumb IPJ	120	N	N	Alive
33	Tirpude et al 2015^40^	50	M	R index nail bed	60	Trauma	Well differentiated	R prox 1/3 middle phalanx	10	N	N	Alive
34	Van Rengen and Degreef, 1996^41^	46	F	L dorsum middle and ring fingers, 2-4th webspaces	6	Epidermolysis bullosa dystrophica of Hallopeau–Siemens	Well differentiated	L middle and ring MCPJ	1.5	N	N	Dead Pneumonia, sepsis, cachexia
35	Virgili et al, 2001^42^	53	F	R middle finger nail bed	36	Chronic infection	Poorly differentiated	R middle finger DIPJ	12	N	N	Alive
36	Virgili et al, 2001^42^	66	M	L thumb proximal nail fold	24	Trauma	Poorly differentiated	L thumb IPJ	12	N	N	Alive
37	Yip et al, 2000^43^	58	F	R thumb nail bed	36	Trauma, chronic infection	Desmoplastic	R thumb IPJ	76	N	N	Alive
38	Yip et al, 2000^43^	60	F	R index nail bed	24	Nil	ND	R index DIPJ	60	N	N	Alive
39	Zabawski et al, 2001^44^	47	F	R middle finger nail bed	ND	HPV	ND	R middle finger DIPJ	1	N	N	Alive

ND – Not documented; DIPJ – Distal Interphalangeal Joint; PIPJ – Proximal Interphalangeal Joint; IPJ – Interphalangeal Joint; MCPJ – Metacarpophalangeal Joint; MC – Metacarpal; CMCJ – Carpometacarpal Joint

**Figure 6:**
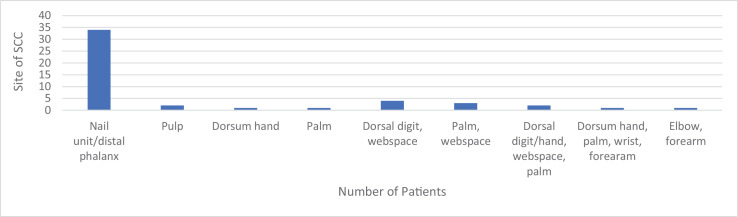
Showing frequency and site of SCCs from case series and literature review.

The right upper extremity was involved in 28 lesions (57.1%) and the left upper extremity in 20 lesions (40.8%); laterality was not recorded for one SCC (2%). There were 34 SCCs involving the nail unit/distal phalanx (69.3%), two involving the pulp (4.1%), one involving dorsum of hand (2%), one involving the palm alone (2%), four involving dorsal digit and webspace (8.2%), three involving palm and webspace (6.1%), two involving dorsal digit/hand, webspace and palm (4.1%), one involving dorsum of hand, palm, wrist and forearm (2%) and one involving elbow and forearm (2%) (Figure 6). Askari et al, Raynor and Schiavon et al report data from SCC exclusively involving the hand.^13^, ^14^, ^15^ Unfortunately, all three articles categorise tumour site slightly differently. However, common to all, including the findings in this review, SCC involving dorsum of the hand and digit occurs most frequently. Webspace and palmar SCC is least common and Raynor describes this as the ‘danger zone’ of the hand; this will be expanded on later in the discussion.^15^ None of the papers have mentioned SCC of the nail unit. It is not clear if they have not included nail unit SCCs or if it has been incorporated into the ‘digital’ category. The authors feel that the nail unit should be viewed as a separate entity as the risk factors for developing SCC at this site differ from those on the proximal region of the digit. Furthermore, rates of recurrence and metastasis appear to differ when compared to SCC involving other parts of the hand.

Length of time from symptoms to diagnosis of SCC ranged from 0.5 months to 204 months (mean 47.4 months). The shortest delay in diagnosis occurred in a patient with a thumb pulp lesion initially thought to be an epidermal cyst and the longest delay arose in a patient with SCC of the nail bed. Ungual SCCs are frequently misdiagnosed as verruca vulgaris, pyogenic granuloma, onycomycosis or paronychia. Due to the ambiguous presentation of nail unit pathology, delay in diagnosis of nail unit SCCs is often the rule rather than the exception.

Risk factors identified from the case series and literature included being Caucasian, UV exposure, smoking, scleroderma, immunosuppression, previous skin cancer, chemical exposure, Human Papilloma Virus, trauma/burns, epidermolysis bullosa, chronic infection and radiation exposure (Table 3). Unfortunately, lack of documentation of risk factors occurred in a number of cases and therefore it is likely that these have been underreported.

**Table 3:** Showing frequency of risk factors for SCC from our case series and literature review.

Risk factor	Number of patients
Caucasian	9
UV exposure	5
Smoking	3
Scleroderma	1
Immunosuppressed	3
Previous skin cancer or in-situ disease	4
Human Papilloma Virus	3
Chemical Exposure	1
Trauma / burn	11
Epidermolysis Bullosa (Inc Kindler Syndrome)	3
Chronic Infection	5
Ionising Radiation Exposure	1
Not documented	7

It is beyond the scope of this review to discuss the pathophysiology of how all risk factors cause SCC however, there are important considerations to be made in view of Human Papilloma Virus (HPV) infection. A number of studies have shown association of HPV with cutaneous SCC.^45^ Alam et al report 72 cases of HPV-associated digital/nail unit SCC and suggest possible genital-digital spread as a mechanism for tumour genesis.^46^ Thus, when nail bed/digital SCC is suspected, patient history must determine previous HPV exposure such as genital warts and HPV cervical cancer not only in the patient but also in their sexual partners. Interestingly, they report that the rate of recurrence of SCC affecting the nail unit/digit to be substantially greater than that of other cutaneous SCCs following tumour clearance.^46^ It has been proposed that although Moh's surgery is beneficial for achieving tumour clearance with digital salvage, residual post-surgical HPV could be responsible for the higher rate of recurrence. Of course, biopsy specimens with PCR-based detection of HPV could be performed alongside that of Moh's surgery to remove both tumour and HPV infection. Undoubtedly, this would be extremely labour intensive and costly given that there is no clear evidence of causality but only an association of HPV and SCC. Additionally, access to clinicians trained in Moh's surgery may be limited as well facilities suiyse for HPV testing. Wide local excision and skin grafting is also an option in these patients however poor cosmesis, stiffness and lack of a graftable bed following tumour extirpation may be limiting factors in some instances. In view of this, patients should be counselled appropriately and informed that close follow up should be observed alongside that of self-surveillance for recurrence or indeed signs of metastasis. After being given these details a small proportion of patients may opt for amputation of the distal phalanx to decrease the risk of recurrence. Fortunately, rates of metastasis from HPV associated periungual SCC are very rare.^46^, ^47^

Histopathological analysis of the specimens reported 9 SCCs to be well differentiated, 12 moderately differentiated, 3 poorly differentiated and one was desmoplastic; there was no documentation of differentiation in the remaining 24 SCCs. Other prognostic factors such as depth of lesion, diameter of lesion and extent of perineural invasion would have added further strength to this study; unfortunately, these factors were absent in most cases.^48^

**Figure 7:**
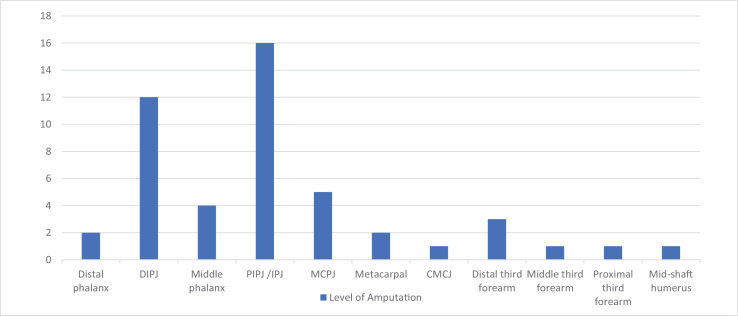
Showing number of patients requiring amputation at a specified level from case series and literature review.

Amputation occurred at multiple levels: two patients underwent amputation through the distal phalanx (4.1%), there were 12 amputations through the DIPJ (24.5%), four through the middle phalanx (8.2%), 16 through the PIPJ/IPJ (32.7%), five through the MCPJ (10.2%), two through the metacarpal (4.1%), one at the level of the CMCJ (2%), three through the distal third of the forearm (6.1%), one through mid-forearm (2%), one through the proximal third of the forearm (2%) and one through mid-shaft of the humerus (2%) (Figure 7).

Length of follow up ranged from 1 month to 120 months (mean 30.5 months), however five cases identified from literature review did not quantify follow-up duration. Unfortunately, due to a low number of patients and lack of follow-up length, we can only report trends in data regarding recurrence, metastasis and mortality rates.

Of the 49 SCCs which were collated from our case series and literature review, rates of recurrence and metastasis were 8.2% (four patients) and 14.3% (seven patients) respectively. The four patients that presented with recurrence all then progressed to metastatic disease; two of these patients died from metastatic disease. When discussing these patients in more detail, they will be referred to as patients one to four.

Patient one was a 74-year-old man that presented with a rapidly growing nodular lesion on his distal palm. He underwent ray amputation of index and middle fingers at the level of the mid metacarpal shaft. Unfortunately, metastatic spread ensued despite radical radiotherapy and he died ten months later. This indicates the aggressive nature of his disease.^17^

The second patient that died with recurrence and metastasis presented with a lesion on the dorsal aspect of the DIPJ and proximal nail fold and was shown to be associated with HPV type 16.^18^ He underwent Moh's micrographic surgery and achieved tumour clearance, however, recurrence of the SCC occurred nine months after the surgery; presence of HPV-16 was again detected. Unfortunately, several months later the patient presented with lymphadenopathy and died of pulmonary complications. This is unusual as metastasis from nail unit SCCs is rare.^18^, ^49^

Patient three was a 23-year-old female with Kindler Syndrome; an autosomal recessive genodermatosis characterised by blistering, photosensitivity and poikiloderma. Actinic keratoses tend to arise in early life and SCC is common due to DNA reparation defects. This patient presented with a palmar SCC however despite ray amputation of index and middle fingers and subsequent below elbow amputation, recurrence and metastasis ensued rapidly. Unfortunately, following amputation and axillary lymphadenectomy the authors report no further on her progress. Her prognosis nevertheless was extremely grave as her SCC measured 8 × 6cm in diameter at the time of presentation, progression of disease was rapid and radiotherapy and aggressive chemotherapy was contraindicated as she suffered from Kindler Syndrome.^22^

The fourth patient with recurrence and metastasis developed SCC on the ring finger pulp. He was a dentist and was exposed to repeated doses of ionising radiation throughout the early stages of his professional career. Interestingly, amputation through the middle phalanx, left axillary surgical debulking and adjuvant chemoradiotherapy kept him free of recurrence and metastasis for a further 6 years.^27^

There were three patients that did not present with local recurrence but instead with SCC metastatic disease. One patient had recessive dystrophic epidermolysis bullosa and died of widespread metastatic disease within one year following amputation.^21^ The second and third patient had a palmar SCC and a dorsal digit/webspace SCC respectively.^28^, ^36^ Both underwent axillary lymphadenopathy and are alive 1.5 and 108 months later.

Total mortality was 14.3% (seven patients) however four deaths were due to unrelated causes and therefore mortality due to metastatic SCC was 6.1% (as discussed above).

In general, 5-year recurrence and metastasis rates for SCC of the hand are greater than that of cutaneous SCC. A review article of primary cutaneous SCC involving all body sites by Alam and Ratner report recurrence rates of 8% and metastatic rates of 5% at 5 years.^46^ Schaivon et al report recurrence rates of 22% at 9 years and a metastatic rate of 28% at 10 years following wide local excision or amputation of SCCs involving the hand.^13^ Askari et al report recurrence rates of 50% at 10 years and metastasis rates of 2% at 20 years, however, these results were based on a range of SCC treatment options.^14^ Lecerf et al specifically looked at nail unit SCC and report a recurrence rate of 30.6% but had no patient with metastasis.^49^

We have no reported cases of recurrence or metastasis to date from our case series. Furthermore, despite there being a short follow-up period for some patients in this review, rates of recurrence and metastasis are in keeping with those stated in current literature reporting a higher rate of recurrence and metastasis from SCCs involving the hand when compared to SCCs of other anatomical sites.

Raynor looked at 273 patients with SCC of the hand. He concluded that the dorsal surface of the proximal phalanx and webspace to be the ‘danger zone’ of the hand as this conferred to SCCs of a more aggressive or complicated nature.^15^ Additionally, four of the seven recorded cases of palmer SCC in his study were also noted to be aggressive.

There were 11 patients (with 11 lesions) with palmar and/or webspace SCCs from this case series and literature review. Analysis of data shows a recurrence and metastatic rate of 18.2% (two patients) and 36.4% (four patients) respectively and mortality (due to SCC metastasis) was 9% (one patient). Comparatively, there were 34 patients with 38 non-webspace or palmar SCCs in this review and recurrence and metastatic rates were 5.3% (two patients) and 7.9% (three patients) respectively; mortality (due to SCC metastasis) was 5.3% (two patients). Of the four patients that had palmar and/or webspace involvement in our case series no-one has had recurrence or metastasis following amputation however follow-up period ranged from 1–48 months.

## Conclusion

This review illustrates that rates of recurrence and metastasis for SCCs affecting the hand are higher than that of general cutaneous SCC. This is true despite amputation of the affected region and the relatively short follow-up period in some cases. Important points to note are that SCCs involving the nail unit have a high rate of recurrence despite tumour clearance due to the possibility of post-operative residual HPV. Fortunately, metastasis from nail unit SCCs is low. Palmar and webspace SCCs are more aggressive than SCCs affecting other parts of the upper extremity and biopsy should be undertaken for any suspicious or non-healing lesion in these regions at the earliest opportunity. Digit/limb preservation and good function is an important consideration; however, this may not be achievable following wide excision of the SCC. With this in mind it is imperative to have a multidisciplinary approach in all complex cases of the SCC involving the upper extremity.

## Conflict of interest

None.

## Funding

None.

## References

[1] Non-melanoma skin cancer in England, Scotland, Northern Ireland, and Ireland, NCIN Data Briefing. April 2013. http://www.ncin.org.uk/publications/data_briefings/non_melanoma_skin_cancer_in_england_scotland_northern_ireland_and_ireland [Accessibility verified, February 13, 2018].

[2] Ilyas E.N., Leinberry C.F., Ilyas A.M. (2012). Skin cancers of the hand and upper extremity. Am Soc Surg Hand.

[3] Osterlind A., Hou-Jensen K., Moller Jensen O. (1988). Incidence of cutaneous malignant melanoma in Denmark 1978–1982. Anatomic site distribution, histologic types and comparison with non-melanoma skin cancer. Brit J Cancer.

[4] Nasser N. (2004). Epidemiologia dos cânceres espinocelulares – Blumenau (SC) – Brasil, de 1980 a 1999. Anais Brasileiros de Dermatologia.

[5] Subramaniam P., Olsen C.M., Thompson B.S. (2017). Article Information anatomical distributions of basal cell carcinoma and squamous cell carcinoma in a population-based study in Queensland, Australia. JAMA Dermatol.

[6] Preston D.S., Stern R.S. (1992). Nonmelanoma cancers of the skin. N Engl J Med.

[7] Johnson T.M., Rowe D.E., Nelson B.R., Swanson N.A. (1992). Squamous cell carcinoma of the skin (excluding lip and oral mucosa). J Am Acad Dermatol.

[8] Dai M., Clifford G.M., Le Calvez F. (2004). Human papillomavirus type 16 and TP53 mutation in oral cancer: Matched analysis of the IARC multicenter study. Cancer Res.

[9] Wang J., Aldabagh B., Yu J., Arron S.T. (2014). Role of human papillomavirus in cutaneous squamous cell carcinoma: A meta-analysis. J Am Acad Dermatol.

[10] Jensen P., Hansen S., Møller B. (1999). Skin cancer in kidney and heart transplant recipients and different long-term immunosuppressive therapy regimens. J Am Acad Dermatol.

[11] Rowe D.E., Carroll R.J., Day C.L. (1992). Prognostic factors for local recurrence, metastasis, and survival rates in squamous cell carcinoma of the skin, ear, and lip: implications for treatment modality selection. J Am Acad Dermatol.

[12] Czarnecki D., Staples M., Mar A. (1994). Metastases from squamous cell carcinoma of the skin in southern Australia. Dermatology.

[13] Schaivon M., Mazzoleni F., Chiarelli A., Matano P. (1988). Squamous cell carcinoma of the hand: Fifty-five case reports. J Hand Surg.

[14] Askari M., Kakar S., Moran S.L. (2013). Squamous cell carcinoma of the hand: A 20-year review. J Hand Surg.

[15] Rayner C.R.W. (1981). The results of two hundred and seventy-three carcinomas of the hand. Hand.

[16] Abner S., Redstone J., Chowdhry S. (2011). Synchronous squamous cell carcinoma in multiple digits. ePlasty.

[17] Ağir H., Adams B.M., Mackinnon C.A. (2007). Squamous cell carcinoma of the palm: A case report. Acta Orthop Traumatol Turc.

[18] Alam M., Caldwell J.B., Eliezri Y.D. (2003). Human papillomavirus-associated digital squamous cell carcinoma: Literature review and report of 21 new cases. J Am Acad Dermatol.

[19] Alghamdi I., Robert N., Revol M. (2016). Fingertips squamous cell carcinoma: Treatment outcomes with surgical excision and full thickness skin graft. Annales de chirugie plastique esthetique.

[20] Batalla A., Feal C., Roson E., Posada C. (2014). Subungual squamous cell carcinoma: A case series. Ind J Derm.

[21] Bosch R.J., Gallardo M.A., Ruiz del Portal G. (1999). Squamous cell carcinoma secondary to recessive dystrophic epidermolysis bullosa: Report of eight tumours in four patients. J Eur Acad Derm Venereology.

[22] Cardin-Langlois E., Hanna D., St-Amant M., Croteau F. (2010). Invasive squamous cell carcinoma of the hand in a patient with kindler syndrome: Case report and literature review. Can J Plast Surg.

[23] Choughri H., Villani F., Sawaya E., Pelissier P. (2011). Atypical squamous cell carcinoma of the nail bed with phalangeal involvement. J Plast Surg Hand Surg.

[24] Figus A., Kanitikar S., Elliot D. (2006). Squamous cell carcinoma of the lateral nail fold. J Hand Surg.

[25] Fino P., Spagnoli A.M., Ruggieri M. (2015). Bilateral hand squamous-cells carcinoma in patient affected with non-Hodgkin lymphoma. Case Rep Lit Rev Il Giornale di Chirurgia..

[26] Fisher J., Masson J., Rosen R. (2006). Squamous cell carcinoma of the hand masquerading as a cutaneous infection. Australas J Derm.

[27] Foley S.J., Pay A., Howell G.P., Holt S. (1995). Metastatic squamous cell carcinoma of the hand and review of the literature. J Royal Army Med Corps.

[28] Gonzalez-Sosa D., Brea-Garcia B., Couto-Gonzalez I., Taboada- Suarez A. (2014). Moderately differentiated squamous cell carcinoma of the palm: An extremely infrequent tumour. BMJ Case Rep.

[29] Grootenboers D.A.R.H., Poortmans P.M.P., Haas R.L.M. (2007). Radiotherapy preserves fingers in the management of subungual squamous cell carcinoma, obviating the need for amputation. Radiother Oncol.

[30] Inkaya E., Sayit E., Tanrivermis Sayit A.T. (2015). Sunungual squamous cell carcinoma of the third finger with radiologic and histopathologic findings: A case report. J Hand Microsurg.

[31] Obiamiwe P.E., Gaze N.R. (2001). Subungual squamous cell carcinoma. Brit J Plast Surg.

[32] Ogawa B., Chen M., Margolis J. (2006). Marjolin's ulcer arising at the elbow: A case report and literature review. Hand.

[33] Olaoye I.O., Adesina M.D. (2013). Subungual squamous cell carcinoma: Report of two cases. Internet J Surg.

[34] Peterson S.R., Layton E.G., Joseph A.K. (2004). Squamous cell carcinoma of the nail unit with evidence of bony involvement: A multidisciplinary approach to resection and reconstruction. Am Soc Dermatol Surg.

[35] Sakamoto A., Shiba E., Hisaoka M. (2015). Squamous cell carcinoma arising from an epidermal cyst of the thumb. Int J Case Rep.

[36] Sanchez T., Walder D., Esenwein P. (2014). Squamous cell carcinoma in combination with symbrachydactyly: Initial management and long term follow–up. Case Rep Ortho.

[37] Shapiro L., Baraf C.S. (1970). Subungual epidermoid carcinoma and keratoacanthoma. Cancer.

[38] Tabesh H., Wilson S., Leonard A.G. (2003). Subungual squamous cell carcinoma. Eur J Plast Surg.

[39] Tambe S.A., Patil P.D., Saple D.G., Kulkarni U.Y. (2017). Squamous cell carcinoma of the nail bed: The great mimicker. J Cutan Aesthet Surg.

[40] Tirpude B.H., Bhanakar H., Shamkuwar A., Gajbhiye A. (2015). Squamous cell carcinoma of the nail bed. Case Rep Int Surg J.

[41] Van Rengen A., Degreef H. (1996). Epidermolysis bullosa dystrophica of Hallopeau–Siemens and squamous cell carcinoma: A case report. Dermatol.

[42] Virgili A., Zampino M.R., Bettoli V., Chiarelli M. (2001). Squamous cell carcinoma of the nail bed: A rare disease or only misdiagnosed?. Acta Derma Venereol.

[43] Yip K.M., Lam S.L., Shee B.W. (2000). Subungual squamous cell carcinoma: Report of 2 cases. J Formos Med Assoc.

[44] Zabawski E.J., Washak R.V., Cohen J.B. (2001). Squamous cell carcinoma of the nail bed: Is finger predominance another clue to etiology? A report of 5 cases. Cutis..

[45] Wang J., Aldabagh B., Yu J., Arron S.T. (2014). Role of human papillomavirus in cutaneous squamous cell carcinoma: A meta-analysis. J Am Acad Derm.

[46] Alam M., Ratner D. (2001). Cutaneous squamous cell carcinoma. N Eng J Med.

[47] McHugh R.W., Hazen P., Eliezri Y.D., Nuovo G.J. (1996). Metastatic periungual squamous cell carcinoma: Detection of human papilloma virus type 35 RNA in the digital tumour and axillary lymph node metastases. J Am Acad Dermatol.

[48] Cherpelis B.S., Marcusen C., Lang P.G. (2002). Prognostic factors for metastasis in squamous cell carcinoma of the skin. Dermatol Surg.

[49] Lecerf P., Richert B., Theunis A., Andre J. (2013). A retrospective study of squamous cell carcinoma of the nail unit diagnosed in a Belgian hospital over a 15-year period. J Am Acad Dematol.

